# Differential effects of small extracellular vesicles from head and neck cancer patients on dendritic cell functions

**DOI:** 10.3389/fonc.2025.1680167

**Published:** 2026-01-14

**Authors:** Diana Huber, Sai Gopal Krishna Yerneni, Linda Hofmann, Sonja Ludwig, Thomas K. Hoffmann, Barbara Wollenberg, Theresa L. Whiteside, Marie-Nicole Theodoraki

**Affiliations:** 1 Department of Otorhinolaryngology, Head and Neck Surgery, Ulm University Medical Center, Ulm, Germany; 2 Department of Laboratory Medicine and Pathology, Mayo Clinic, Rochester, MN, United States; 3 Department of Molecular Pharmacology and Experimental Therapeutics, Mayo Clinic, Rochester, MN, United States; 4 Department of Otorhinolaryngology, Head and Neck Surgery, University Hospital Mannheim, Medical Faculty Mannheim, University of Heidelberg, Mannheim, Germany; 5 Department of Otorhinolaryngology, Head and Neck Surgery, Klinikum rechts der Isar, Technical University Munich, Munich, Germany; 6 University of Pittsburgh Medical Center (UPMC) Hillman Cancer Center, University of Pittsburgh School of Medicine, Pittsburgh, PA, United States

**Keywords:** head and neck cancer, HNSCC, sEVs, tumor-derived sEVs, dendritic cells, antigenprocessing machinery, HPV - human papillomavirus

## Abstract

**Background:**

Tumor-derived small extracellular vesicles were shown to contribute to immunosuppression in various cancer types, including head and neck squamous cell carcinoma. Dendritic cells, major regulators of specific anti-tumor immune response, are often impaired in their function in the context of cancer.

**Methods:**

Monocyte-derived dendritic cells were incubated with isolated small extracellular vesicles during or after maturation. Cell surface markers and antigen processing machinery were assessed by flow cytometry. Vesicle-treated dendritic cells were tested for interleukin 12p70 production by ELISA, and differences in uptake of labeled vesicles were shown by confocal microscopy.

**Results:**

Small extracellular vesicles derived from HPV(+) tumor cell lines increased maturation and activation of immature dendritic cells, priming them for more rapid phagocytosis. Immature dendritic cells co-incubated with vesicles had lower levels of antigen presenting machinery components and produced less IL12p70 than controls, while similarly co-incubated mature dendritic cells remained unaffected. Vesicles in plasma of patients with head and neck cancer inhibited functions of mature dendritic cells by downregulating expression of antigen presenting machinery components.

**Conclusion:**

Our results uncover mechanisms through which tumor-derived small extracellular vesicles modulate dendritic cell functions locally and systemically. These mechanisms may also affect dendritic cell-based vaccine efficacy.

## Graphical Abstract

**Figure:**
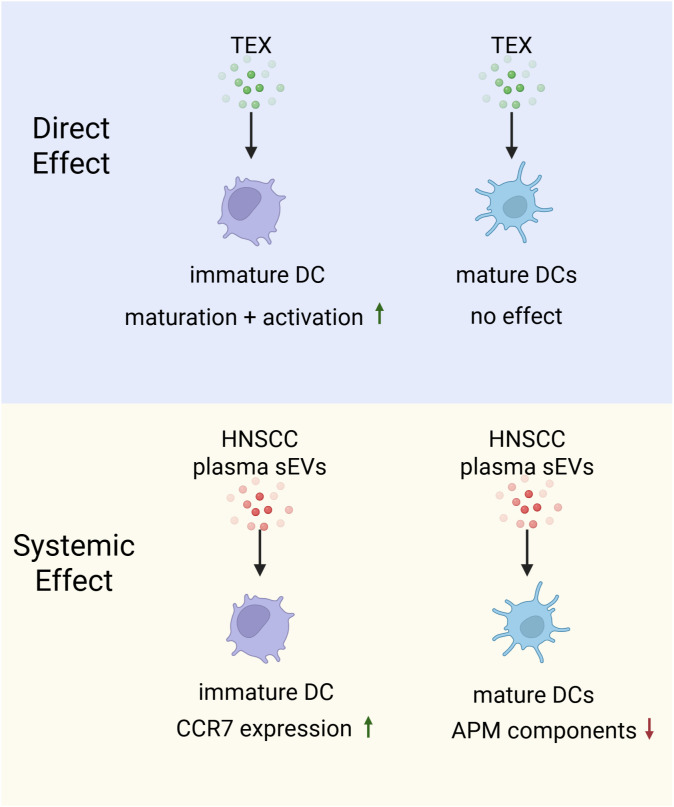

## 1 Introduction

Head and neck squamous cell carcinoma (HNSCC) is a highly immunosuppressive malignancy, which develops following an infection with the human papilloma virus (HPV) or Epstein-Barr Virus (EBV) or may be induced by tobacco and alcohol abuse (1). Despite significant progress in cancer research, the 5-year survival rate for patients diagnosed with HNSCC has remained very low, especially for HPV(-) patients (2). Therefore, understanding of the tumor-immune system interplay emerges as a key requirement for developing effective therapies, especially for HNSCC patients who fail to respond to existing therapies.

Among various efforts designed to improve therapy, an interesting approach includes dendritic cell (DC) based vaccines, where autologous *ex vivo* activated DCs transfused back to the patient are expected to cross-present tumor-associated antigens (TAAs) to T cells in order to induce an anti-tumor immune response (3). Although DCs are typically present in small numbers in tumors, their involvement in anti-tumor immunity is crucial and their presence is linked to improved anti-tumor response and overall survival of the patients (3–5). Removal of tumor-draining lymph nodes containing anti-tumor DCs, was shown to significantly reduce the anti-tumor immune response and T cell activation, worsen patient survival and responsiveness to immune checkpoint inhibitor treatment, highlighting the importance of functional DCs for cancer therapy (6, 7). To induce tumor-specific immune responses, DCs are indispensable for acquiring, processing, and presenting TAAs on the major histocompatibility complex (MHC) class 1 molecules to activate cytotoxic T cells, which are the primary effectors in recognizing and eliminating cancer cells (8, 9). This process of cross-presentation can either be carried out in the TME or in tumor-draining lymph nodes. However, antigen processing and presentation represents a complex cascade of numerous steps, which need to be performed correctly for adequate interaction with T cells. In the TME, DCs internalize proteins from their surroundings, including antigens derived from necrotic tumor cells, and process these proteins by the antigen-processing machinery (APM). First, internalized proteins are broken down to peptides by the proteasome or immunoproteasome (10). The peptides are then loaded on MHC molecules in the endoplasmic reticulum (ER). Several chaperone proteins (e.g. calreticulin, tapasin and ERp57) and transporters associated with antigen processing-1 and 2 (TAP1 and TAP2) are required for stabilization and mediation of this process. After successful binding of a suitable peptide, the MHC-peptide complex is released from the chaperone proteins and translocated to the cell surface for presentation to T cells (11). For functional antigen presentation, fully matured DCs are necessary (11). Furthermore, DCs must be able to migrate into lymph nodes after stimulation by CCR7 ligands and produce sufficient levels of IL-12p70 to activate helper T cells (TH cells) and cytotoxic T cells to be able to induce an effective anti-tumor immune response (12, 13). However, this activation cue is frequently impaired in cancer (12, 13). Specifically, the process of DC maturation or antigen processing and presentation by DCs is often compromised by the presence of factors produced by the tumor, which prevent its recognition by the immune system. Co-incubation of DCs with tumor cells, for example, leads to suppression of DC ability to cross-present TAAs and induces DC apoptosis (9, 10), which implies that most DCs found in the tumor milieu are immature and non-functional. In addition, DCs, which were activated *ex vivo* for DC based vaccination, were shown to be functionally impaired after being exposed to immunosuppressive factors of the TME, thereby limiting their clinical efficacy (14).

Recent studies suggest that tumor-derived small extracellular vesicles (sEVs), also referred to as TEX play a key role in modulating functions of immune cells in the TME (15–19). These vesicles are heterogenous in size (range from 50 to 150 nm) and contain molecules derived from the parental cell (13). Tumor-derived sEVs or TEX mediate cell-to-cell communication between tumor cells and all other cells in the TME, including immune cells. By interaction with surface receptors on recipient target cells or by delivering their cargo, they induce functional reprogramming that in recipient immune cells leads to suppression of anti-tumor immune responses. We have previously shown that in HNSCC, circulating sEVs contribute to the immunosuppressive characteristic of HNSCC patients and modulate functions of CD8(+) T cells, regulatory T cells or B cells (20–22).

Previous co-culture experiments of melanoma cells with DCs led to decreased expression of antigen processing machinery (APM) components and impaired functions of DCs (23). In those experiments performed in a transwell system, soluble factors were responsible for the observed functional effects. Furthermore, our earlier work illustrated different effects of TEX derived from HPV(+) or HPV(-) HNSCC cell lines on DC activation and expression of APM components. HPV(-) TEX inhibited DC maturation and expression levels of APM components while HPV(+) HNSCC TEX increased expression levels of the maturation markers without significantly affecting expression of APM proteins in iDCs (24). Here, we further investigate whether HPV(+) TEX activate anti-tumor functions in DCs compared to non-HNSCC sEVs. Furthermore, we investigate whether circulating plasma-derived sEVs from HNSCC patients have similar effects to cell culture derived sEVs, revealing potential targets to either inhibit sEV-mediated suppression or enhance TEX-driven activation of DCs. These investigations aim to elucidate whether HNSCC-derived sEVs can serve as an effective source of tumor antigens for dendritic cell-based vaccines.

## 2 Methods

### 2.1 Blood processing for isolation of patient-derived sEVs and PBMC

Venous blood samples were obtained from healthy volunteers or HNSCC patients from the UPMC Otolaryngology Clinic in Pittsburgh, PA, USA. All subjects donating blood specimens for this study signed an informed consent approved by the Institutional Review Board of the University of Pittsburgh (IRB #960279, IRB#0403105, and IRB #0506140).

For subsequent isolation of sEVs, blood plasma was collected by centrifugation of all blood specimens at 1,000 x g for 10 min and plasma was stored frozen at -80 °C in 2mL aliquots.

For functional studies, primary DC cultures were prepared. Peripheral blood mononuclear cells (PBMC) were isolated from buffy coats of healthy donors (HDs) by density gradient centrifugation using Ficoll-Hypaque gradients (GE Healthcare Bioscience, Chicago, USA). PBMCs were washed and processed immediately as described below.

### 2.2 Isolation and cultivation of DCs

To generate DCs, monocytes were isolated by CD14 positive selection using CD14 MicroBeads (#130-050-201, Miltenyi Biotec, Bergisch Gladbach, Germany) according to the manufacturer’s instructions. Briefly, PBMCs were resuspended in MACS buffer (Miltenyi Biotec) containing 0.2% BSA, mixed with 20 µL CD14 MicroBeads per 10^7^ cells and incubated for 15 min at 4 °C. Labeled cells were then separated by magnetic columns, washed and cells were seeded at 0.5 x 10^6^ cells per mL in AIM V Medium supplemented with 1000 U/mL GM-CSF (#130-095-372, Miltenyi Biotec) and 1000U/mL IL-4 (#130-093-917, Miltenyi Biotec) for 6 days, according to the protocol described earlier (23). On day 3, half of the medium was exchanged with new AIM V Medium containing 2000 U/mL GM-CSF and 2000 U/mL IL-4. To generate mature dendritic cells (mDCs), on day 6 half of the medium was removed and replaced by new AIM V Medium containing the maturation factors as described in **Table 1** and incubated for 24 h.

**Table 1:** Supplements for DC maturation.

Maturation factor	Concentration
IL-1β	25 ng/mL
GM-CSF	1000 U/mL
PGE2	10^–6^ mol/L
IL-6	500 U/mL
TNF	50 ng/mL

### 2.3 Cell culture

The Human Papilloma Virus (HPV) positive HNSCC tumor cell line UPCI-SCC-90, originating from the tongue of a 46-year-old white male patient at the University of Pittsburgh, was used for obtaining tumor-derived sEVs (TEX). The THP-1 cell line, a monocyte cell line derived from 1-year-old male acute monocytic leukemia patient and obtained from ATCC was used as non-HNSCC control. The cells were grown in 150 cm^2^ cell culture flasks with 25 mL DMEM (gibco, Thermo Fisher Scientific, Waltham, MA, USA) supplemented with 1% penicillin and streptomycin and 10% fetal bovine serum (FBS) depleted of sEVs by ultracentrifugation. Before sEV isolation, the cells were cultured at 37 °C and 5% CO_2_ for 48 h to72 h.

### 2.4 Isolation of sEVs

sEVs were either isolated from supernatants of the cell lines UPCI-SCC-90 and THP-1 or from the plasma of HNSCC patients and healthy donors (HDs). For isolation of cell line-derived sEVs, conditioned cell culture medium was harvested and concentrated from 50 to 1 mL using Vivacell 100 filter units (MWCO 100,000, Sartorius Corp, Bohemia, NY, USA). For isolation of plasma-derived sEVs, the frozen plasma aliquots were thawed, centrifuged at 2,000 x g for 10 min and at 10,000 x g for 30 min, followed by ultrafiltration through 0.22 µm syringe-driven filters (Merck Millipore, Billerica, MA, USA). For size exclusion chromatography (SEC), 1 mL of concentrated supernatant or plasma was loaded onto sepharose-packed columns (sepharose CL-2B, Cytiva, Uppsala, Sweden) and sEV-containing fraction #4 was collected (25). Protein concentration of the collected fraction was determined by Pierce BCA Protein Assay (#23225, Thermo Fisher Scientific) and adjusted to 100 µg/mL using 100,000 MWCO Vivaspin 500 Centrifugal Concentrators (Sartorius Corp) centrifuged at 2,000 × g for 10–15 min. For functional assays, sEVs were applied to dendritic cells with a final concentration of 10 µg/mL for all sEVs.

### 2.5 Characterization of sEVs

Isolated sEVs were characterized to meet the criteria of the minimal information for studies of extracellular vesicles (MISEV) guidelines (26), as previously described (27). Characterization of cell culture-derived sEVs was shown in our previous work (24), and characteristics of patient-derived sEVs are shown in **Supplementary Figure 1**. Briefly, nanoparticle tracking analysis was performed to show size distribution and number of particles in the sample and morphology was imaged by JEOL 1400 transmission electron microscope (TEM) after negative staining of sEVs with 3% uranyl acetate on carbon-coated copper TEM grids. Absence and presence of specific markers was confirmed by western blot analysis with tetraspanins (CD9, CD63) and TSG101 as positive markers for sEVs and Grp94 and ApoA1 as negative markers, only present in cell lysates or plasma, respectively.

### 2.6 miRNA profiling of sEVs

sEV miRNA content was analyzed by NanoString nCounter technology, as previously described (28). In short, RNA was isolated using the miRNeasy Micro Kit (Qiagen, Hilden, Germany) according to manufacturer’s instructions. The Human v3 miRNA Assay was performed using 500 pg of sEV RNA on the nCounter^®^ SPRINT system (Nanostring Technologies, Seattle, WA, USA) at the nCounter^®^ Core Facility of the University of Heidelberg, Germany. miRNA expression data were analyzed using the nSolver 4.0 software. Raw data were normalized using the positive ligation controls.

### 2.7 Internalization of labeled sEVs by dendritic cells

sEVs were labeled with PKH26 fluorescent cell linker (MINI26, Sigma-Aldrich) diluted 1:50 in the provided Diluent C for 5 min. The staining reaction was stopped by adding 2 mL of ultracentrifuged FBS. Labeled sEVs were washed with PBS using 100K Amicon Ultra 0.5 mL centrifugal filter (EMD Millipore) and co-incubated with DCs for 5min (t0), 15 min and 30 min at 37 °C. The 5 min time point was considered as t0, because no uptake was expected after 5 min. After incubation, cells were washed with stripping buffer (146 g NaCl, 2.5 mL acetic acid, 500 mL dd H_2_O) for 2 min to remove sEVs on the surface, washed 3x with PBS and fixed with freshly prepared 1.6% paraformaldehyde for 20 min. Cells were washed 3x with PBS, permeabilized with 0.1% Triton X for 1 min and stained with Alexafluor488-Phalloidin for F-actin and with Hoechst for nuclei. Stained cells were imaged with the Carl Zeiss LSM 800 confocal microscope.

### 2.8 Experimental design

**Figure 1:**
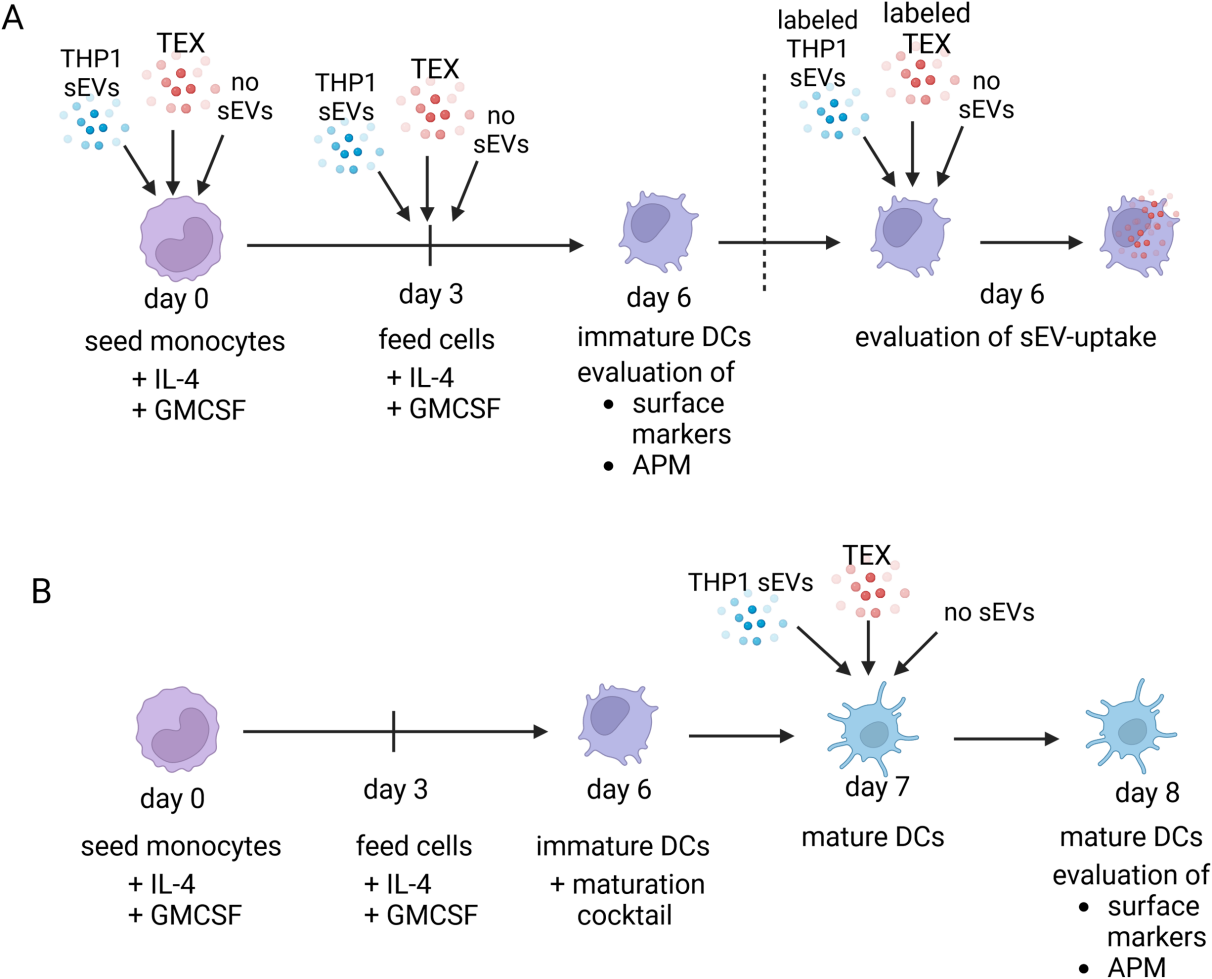
Experimental design for treatment of DCs with TEX derived from the SCC-90 a HNSCC cell line, monocyte cell line (THP1 sEV) or without sEV (no sEV). The same experimental design was used with primary sEV derived from blood plasma of HNSCC patients or healthy individuals. **(A)** Monocytes were seeded and directly incubated with or without sEV on day 0 and day 3 until day 6, when the analysis was performed. **(B)** monocytes were seeded, differentiated and maturated to mature dendritic cells before adding sEV on day 7 for 24h.

sEVs were added in different experimental set-ups, as depicted in **Figure 1**, to investigate the effect of sEVs on expression of APM proteins and activation markers in different maturation stages of DCs. Primary monocytes were differentiated to immature DCs (iDCs) by adding IL-4 and GM-CSF to the culture medium on day 0 and day 3 until monocytes are differentiated to iDCs on day 6. To obtain mature DCs (mDCs), the maturation cocktail, as described above, was added for 24 h. To investigate the effect of sEVs during the differentiation process to iDCs (Design A), monocytes were incubated on day 0 and day 3 with sEVs derived from the HNSCC cell line UPCI-SCC90 (TEX), from the non-HNSCC cell line THP-1 (THP1 sEVs) and with PBS as negative control (no sEVs) (**Figure 1A**). On day 6, after differentiation to iDCs, iDC characteristics were evaluated by flow cytometry, IL12p70 production and uptake of labeled sEVs. In the second experimental design (Design B), the effects of sEVs on already mature mDCs was investigated by differentiation of monocytes to iDCs and adding the maturation cocktail, before sEVs were added on day 7 and mDC characteristics were evaluated on day 8 (**Figure 1B**). To investigate the effect of circulating patient-derived sEVs on iDCs and mDCs, the same experiments were also performed using sEVs derived from HNSCC patients and healthy donors.

### 2.9 Staining for flow cytometry

After incubation with sEVs as per Design A or Design B (**Figure 1**), DCs were detached from the well using trypsin and washed with PBS containing 0.2% BSA. Cells were stained with fluorochrome-labeled antibodies against cell surface markers and co-stimulatory proteins: CD80 (#557227, 10 µL) and CD86 (#555660, 5 µL) purchased from BD Biosciences, CD83 (#IM3240U, 10 µL) and HLA-DR (IM0463U, 5 µL) from Beckmann Coulter and CCR7 (#FAB197E, 10 µL) from R&D Systems to determine the maturation state of DCs. For expression of APM proteins, DCs were permeabilized before performing intracellular staining. Antibodies against TAP1, TAP2, calreticulin, LMP-7, tapasin and ERp57 were kindly provided by Dr. Soldano Ferrone (Harvard University, Boston, MA, USA), who conjugated primary mouse antibodies to APC or FITC using the Lightning-Link kit (Innova Biosciences), as described before (29–31). For all antibodies, matching isotypes were used as controls.

### 2.10 Induction of IL-12p70 production

To evaluate the functions of DCs IL-12p70 production was measured. Immature or mature DCs, pre-incubated with/without sEV were harvested, washed and seeded in 96-well plates (2 x 10^4^ cells per well). DCs were stimulated to produce IL-12p70 by the addition of 5 x 10^4^ CD40L-transfected J558 cells (32, 33). The transfected J558 cells were previously shown to induce activation equivalent to activated CD4+ T cells and soluble CD40L and were used here to mimic interactions of DCs with Th cells (34, 35). After 24 h the amount of produced IL-12p70 was determined by ELISA (Endogen), according to the manufacturer’s protocol. All conditions contained 1,000 U/mL GM-CSF, to maintain cell viability.

### 2.11 Statistical analysis

The flow cytometry data was acquired using Kaluza (v1.5, Beckman Coulter) and analyzed using Graph Pad Prism by displaying means and standard deviation. To test for statistical significance, datasets were first tested for normality by Shapiro-Wilk test and differences were analyzed using ordinary one-way ANOVA for parametric and Kruskal-Wallis for non-parametric data, followed by Holm-Sidak’s multiple comparisons test or Dunn’s multiple comparisons test, respectively. A p-value of 0.05 and less was considered as statistically significant with * representing p < 0.05; **: p < 0.01 and ***: p < 0.005.

## 3 Results

### 3.1 Clinical parameters of sEV donors

For this study sEVs were isolated from 12 HNSCC patients with a mean age of 60,7 years and 3 healthy donors (HD) with a mean age of 59 years at the time of sample acquisition, as summarized in **Table 2**. 91.7% of HNSCC patients and all HDs were male. 83.3% of HNSCC patients were smokers, 16.7% were non-smokers and all HDs were light smokers. The majority of primary tumors were located in the oropharynx and larynx, each accounting for 41.7% of patients, whereas 16.7% of patients had tumors located in the oral cavity. 50% of the patients were tested positive for HPV by PCR for HPV-16 DNA and by immunohistochemistry for p16 and the other 50% were tested negative. 25% of the patients were diagnosed with a tumor size T1, 41.7% with T2, 8.3% with T3 and 25% with T4 and 75% had lymph node metastases (N+).

**Table 2:** Clinical parameters of sEV donors.

Clinical Parameter	HNSCC patients (n = 12)		Healthy donors (n = 3)	
Mean age	60.7		59	
	range: 47-79		range: 40-71	
	n	%	n	%
Gender				
male	11	91.7	3	100
female	1	8.3	0	0
Smoking				
smokers	10	83.3	3	100
non-smokers	2	16.7	0	0
Primary tumor site				
Oropharynx	5	41.7		
Oral Cavity	2	16.7		
Larynx	5	41.7		
HPV status				
positive	6	50.0		
negative	6	50.0		
T status				
T1	3	25.0		
T2	5	41.7		
T3	1	8.3		
T4	3	25.0		
Nodal status				
N0	3	25.0		
N+	9	75.0		

### 3.2 Dynamics of sEV uptake by primed DCs

**Figure 2:**
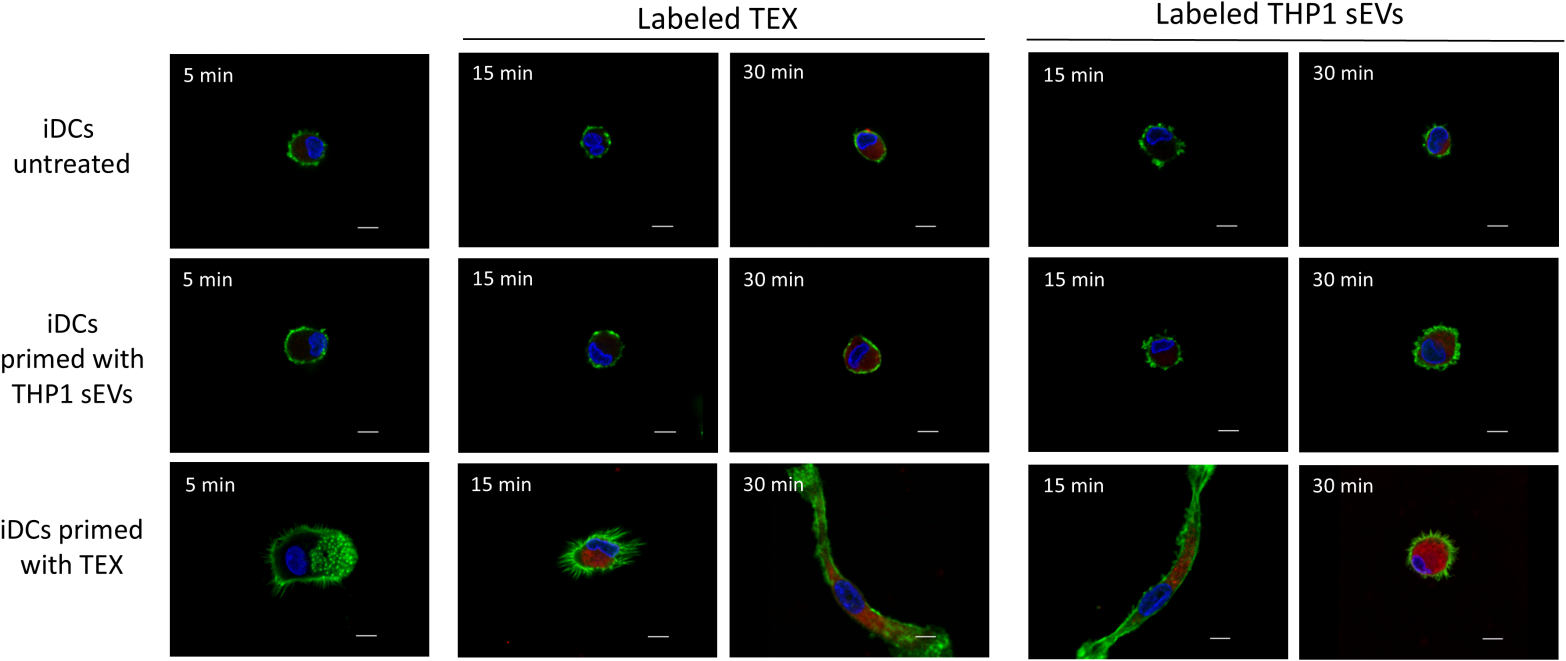
Faster uptake of sEVs by immature dendritic cells (iDCs) after pre-incubation with TEX. DCs were cultivated according to experimental design A and incubated with PKH26-labeled sEVs from the tumor cell line SCC-90 (Labeled TEX) or from THP-1 cell line (Labeled THP1 sEVs). The images are representative from one of 3 experiments with nuclei stained with Hoechst in blue, F-actin in green and labeled sEV in red. Scale bar represents 5 µm.

As professional antigen-presenting cells, DCs are constantly internalizing external peptides present in the microenvironment for processing and cross-presentation to T cells. Here, we investigated the uptake of sEV by DCs. On day 6, iDCs were incubated with labeled sEV derived from the HPV(+) HNSCC cell line SCC-90 (TEX) or from the monocyte cell line THP-1 (THP1 sEV). After 5min of co-incubation, no sEV uptake was expected and this time point was considered as negative control (t0). After 30 min of incubation, untreated iDCs internalized labeled TEX as well as THP1 sEV (**Figure 2**, first row).

IDCs, which were pre-incubated with THP1 sEVs on day 0 and 3 showed the same sEV uptake on day 6 as untreated DCs. Labeled THP1 sEVs were internalized by untreated DCs after 30min. However, DCs pre-incubated with TEX internalized significant amounts of labeled sEVs after 15min of co-incubation. Interestingly, the cell origin of labeled sEVs did not affect the dynamics of uptake by TEX-primed DCs, since THP1 sEVs as well as TEX were internalized after 15min. Furthermore, TEX-primed DCs exhibited an elongated phenotype, which is typically observed after DC activation. This phenotype was only observed in TEX-primed DCs, not in untreated DCs or DCs primed with THP1 sEVs. In summary, TEX primed DCs to efficiently internalize sEVs faster regardless of the sEV origin, and thereby to activate DCs.

### 3.3 Maturation and activation of DCs in presence or absence of TEX

**Figure 3:**
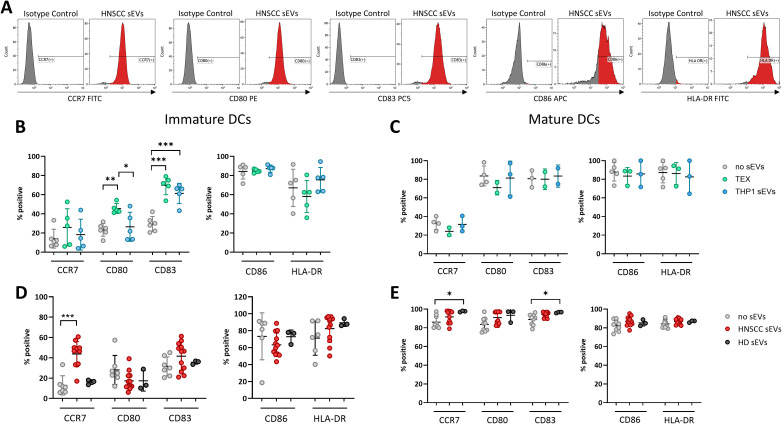
Maturation and activation markers of DCs incubated with sEVs from different origins. **(A)** Representative histograms of DCs incubated with HNSCC sEVs and labeled with conjugated antibodies or isotype controls. **(B–E)** Percentage of positive cells measured by flow cytometry is shown from immature DCs (exp. Design A) after incubation with sEVs from the tumor cell line (TEX) or THP1 sEVs **(B)** or primary sEVs from HNSCC patients (HNSCC sEVs) or healthy donors (HD sEVs) **(D)** and data of mature DCs (exp. Design B) incubated with TEX and THP1 sEVs **(C)** or primary sEVs **(E)**. The data are from experiments performed with DCs generated from monocytes of 2 healthy donors and incubated with sEVs isolated from 12 HNSCC patients and 3 healthy donors or from cell cultures (n=5 for iDCs and n=3 for mDCs). Differences with statistical significance were indicated with stars above the respective comparison with * representing p < 0.05; **: p < 0.01 and ***: p < 0.005. All other comparisons were not significant.

To investigate the effect of TEX on the DC differentiation (from monocytes to iDCs) and confirm activation by TEX, sEVs were added during differentiation on days 0 and 3 (**Figure 1**, design A) and expression of activation and maturation markers in DCs were determined by measuring the percentage of positive cells (**Figure 3A**). Indeed, incubation with TEX during the differentiation process led to increased expression of activation markers CD80 and CD83 (**Figure 3B**), which is in line with the findings of elongated DCs illustrated above. Incubation with sEVs derived from the monocyte cell line, THP-1 (used as control), also induced increased levels of CD83, however, CD80 expression was only upregulated by TEX and not THP-1 sEVs. Measuring effects of sEVs on already mature DCs (design B) revealed no effects of TEX or THP-1 sEVs. Thus, TEX or THP-1 sEVs did not have stimulatory or inhibitory effects on the expression of surface markers after DC maturation (**Figure 3C**).

Addition of plasma-derived sEVs from HNSCC patients (HNSCC sEVs) during DC differentiation (design A, **Figure 3D**) did not lead to the same results observed with TEX (**Figure 3B**). No changes in CD80, CD83, CD86 or HLA-DR were visible compared to untreated DCs or DCs treated with sEVs from plasma of HDs. Interestingly, expression of CCR7 was significantly upregulated after incubation with HNSCC sEVs compared to no sEVs or HD sEVs. Incubation of mature DCs (design B, **Figure 3E**) with HNSCC sEVs did not induce significant changes compared to untreated DCs, as similarly high levels of all surface markers were observed. However, the addition of HD sEVs increased levels of CCR7 and CD83 compared to untreated DCs. Furthermore, discrimination of HNSCC patients according to their HPV status showed no differences in the expression of surface markers (**Supplementary Figures 2**, **3**), therefore no further HPV-dependent separation of patient-derived sEVs has been performed. Overall, these findings suggest that TEX can pre-activate DCs, which are undergoing differentiation, whereas matured DCs are much less responsive to TEX from cell culture supernatants or those present in patients’ plasma.

### 3.4 Effect of sEVs on the antigen processing machinery

**Figure 4:**
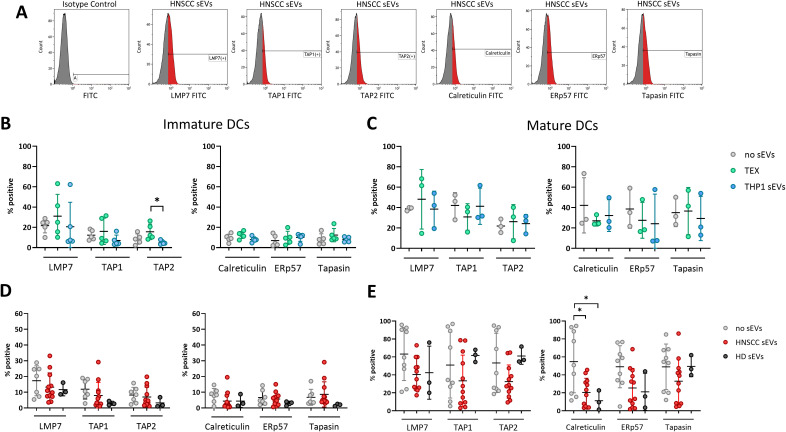
Decreased expression of APM components after incubation with HNSCC sEVs. **(A)** Representative histograms of DCs incubated with HNSCC sEVs and labeled with conjugated antibodies or isotype control. **(B-E)** Percentage of expression of APM components after incubation of iDCs with TEX and THP1 sEVs **(B)** or HNSCC and HD sEVs **(D)** or incubation of mDCs with TEX **(C)** or patient-derived sEVs **(E)**. The data are from experiments performed with DCs generated from monocytes of 2 healthy donors and incubated with sEVs isolated from 12 HNSCC patients and 3 healthy donors or from cell cultures (n=5 for iDCs and n=3 for mDCs), as in **Figure 3**. Differences with statistical significance were indicated with stars above the respective comparison with * representing p < 0.05. All other comparisons were not significant.

To induce targeted anti-tumor immunity, DCs must present TAAs to cognate T cells. Here we investigated whether TEX have an impact on the APM, by measuring expression of six markers involved in antigen processing (LMP7, TAP1, TAP2, tapasin, calreticulin and Erp57) by flow cytometry (**Figure 4A**). Co-incubation of iDCs with TEX did not induce any significant changes in the expression of markers compared to untreated DCs (**Figure 4B**). Only TAP2 expression was significantly increased after incubation with TEX compared to THP1 sEVs. As expected, levels of proteins involved in antigen processing were generally lower in iDCs compared to mDCs. In mDCs, co-incubation with any sEVs did not induce significant changes in APM marker expression (**Figure 4C**).

Similarly, in iDCs incubated with plasma-derived sEVs the levels of APM components were not affected by sEV incubation (**Figure 4D**). In mature DCs incubated with HNSCC sEVs, calreticulin was significantly decreased compared to untreated DCs (**Figure 4E**), but also with HD sEVs. Furthermore, the other APM components were also slightly decreased by HNSCC sEV incubation, however not significantly, because of the high variability of the data.

Decreased APM expression in mature DCs is associated with immune tolerance and immune tolerance in DCs has been reported in the literature to be induced by miR-212-3p from pancreatic cancer cell-derived sEVs (36). In an independent study, we analyzed the miRNA profiles of plasma-derived sEVs from HDs and HNSCC patients. Notably, also in our data miR-212-3p levels were significantly elevated in HNSCC sEVs compared to HD sEVs, as shown in **Supplementary Figure 1E**.

### 3.5 Functions of DCs

**Figure 5:**
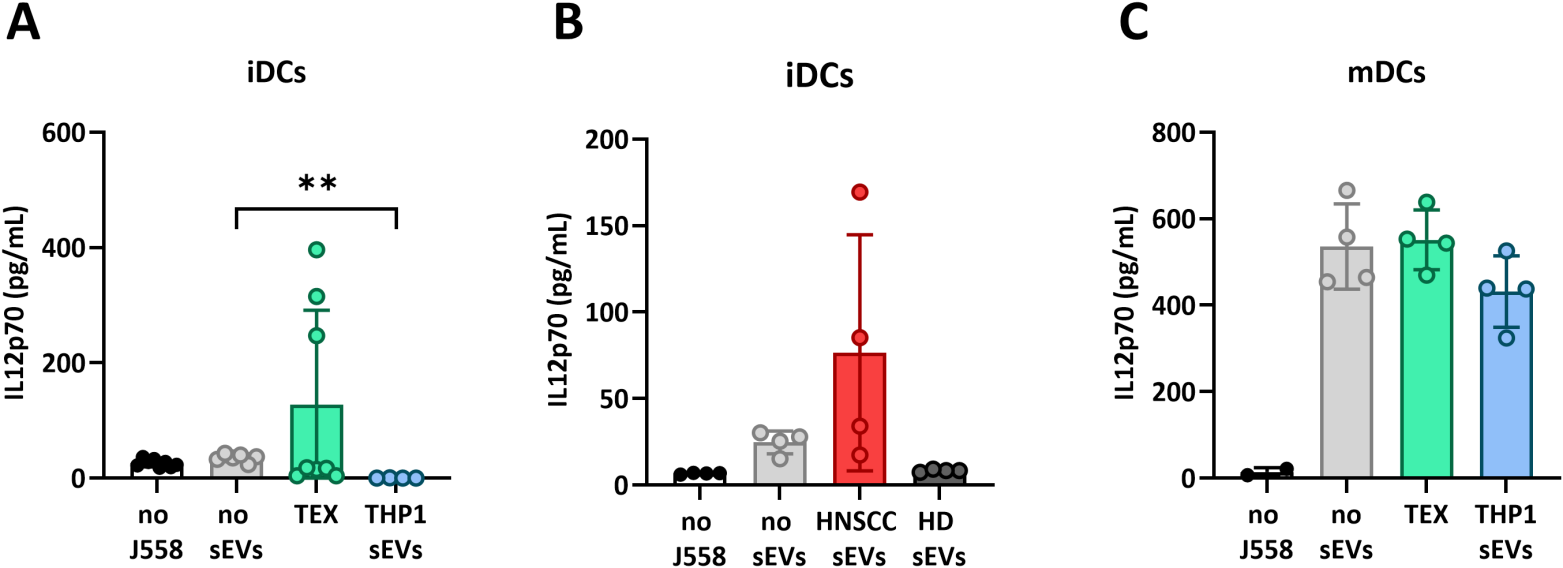
Levels of IL12p70 produced by DCs after activation by J558 transfected T cells. (A+B) iDCs incubated with TEX **(A)** or HNSCC sEVs from plasma **(B)**. **(C)** mDCs incubated with TEX. The data are from experiments performed with DCs generated from monocytes of 2 healthy donors and incubated with sEVs isolated from 4 HNSCC patients and 3 healthy donors or from cell cultures. Differences with statistical significance were indicated with stars above the respective comparison with * representing p < 0.05 and **: p < 0.01. All other comparisons were not significant.

For functional evaluation of DC activation and responsiveness to stimulation by Th cells, pre-treated DCs were co-incubated with CD40L-transfected J558 cells (used as surrogates for activated CD4(+) T cells) and IL12p70 production by DCs was measured in the supernatant. As expected, untreated iDCs produced low levels of IL-12p70 (**Figures 5A, B**) with levels only around 40 pg/mL compared to levels of approximately 500 pg/mL in untreated mDCs (**Figure 5C**). Pre-incubation with THP1 sEVs or HD sEVs further decreased levels of IL-12p70 in iDCs, while pre-incubation with TEX increased IL12-p70 production in some iDCs up to 400 pg/mL, but decreased IL-12p70 production levels in other iDCs, similar to THP1 sEVs (**Figure 5A**). sEVs from HNSCC patients’ plasma also increased production of IL-12p70 in some iDCs, but not in others. In contrast, IL-12p70 production in mDCs was not affected by sEVs (**Figure 5C**). These results suggest that coincubation of iDCs with TEX and HNSCC sEVs results in variable activation of iDC functions that may be dependent on DCs differentiation level as well as sEV quality and quantity.

## 4 Discussion

Tumor-derived sEVs were previously shown to inhibit maturation of DCs by several groups of investigators (37–40). However, in this study and our previous work (24), incubation of DCs with sEVs from an HPV(+) HNSCC cell line has led to upregulation of DC maturation and/or expression levels of APM in DCs. This observation was specific for HPV(+) sEVs, as incubation with HPV(-) HNSCC sEVs decreased expression of activation markers in our previous study (24). The present study was designed to further investigate the effects of HPV(+) HNSCC sEVs on DC phenotype and function, which might be helpful for *ex vivo* DC activation. We investigated the effects induced by circulating, plasma-derived sEVs of HNSCC patients, regarding their role in systemic immunosuppression or as potential candidate for patient-personalized cancer vaccination. The latter experiments were performed to gain insights into systemic effects mediated by sEVs, imitating the effects of sEVs in the periphery, while cell line-derived sEVs represent direct effects of tumor sEVs in the TME.

As professional APCs, DCs are highly efficient at taking up exogenous factors in their microenvironment, including sEVs as evidenced by our data. Pre-incubation of iDCs with TEX increased this uptake and has led to activation of iDCs by alterations in the shape (elongation) of DCs and increased expression of CD83 and CD80 on their surface. TEX from HPV(+) cells were previously shown to carry HPV-associated proteins and RNA (24, 41, 42), which may contribute to their stimulatory effects on DCs (24). Furthermore, tumors in HPV(+) HNSCC patients are typically associated with active inflammation and tumors are considered as “immunologically hot” tumors. These tumor features are thought to lead to a better response to therapy and overall survival of HPV(+) patients (43). Although TEX were often reported to inhibit DC differentiation and function, there is also evidence, that TEX carry tumor-associated antigens (TAAs), which promote anti-tumor immune responses (44). This has served as a rationale for *ex vivo* DC activation by pre-incubating them with EVs from cancer patients’ plasma or from cancer cell cultures, showing promising results (45, 46). However, the inhibitory components present in most TEX compromise the effectiveness of this strategy. Since HPV(+) TEX seem to have a lower inhibitory potential than HPV(-) TEX, this approach might be suitable for HPV(+) HNSCC patients, highlighting the need for tailored vaccination-based therapeutic approaches, depending on the HPV-status of the patient.

The effects of TEX isolated from cultured HNSCC cells are relatively uniform and can be directly attributed to tumor cells. In contrast, plasma-derived sEVs have diverse cellular origins and induce a variety of changes in recipient cells. While tumor cells are significant TEX producers, other cells in contact with the blood stream, including immune cells or epithelial cells, also contribute to the pool of circulating sEVs (47). Thus, while TEX-induced changes in DCs reflect tumor cell interactions with DCs, sEVs in plasma reflect systemic reprogramming of immune and non-immune cells in tumor patients. In contrast to cell line-derived sEVs, plasma sEVs did not show significant differences in expression of DC maturation and activation markers between HPV(+) and HPV(-) patients. This is likely attributable to the heterogeneous composition of plasma EVs, whereby additional cellular contributions may mask HPV-related differences. In line with this hypothesis, not only TEX were shown to be relevant for reprogramming immune cells in tumor patients, but also EVs from other cellular origin, including immunosuppressive immune cells (48). HNSCC is a highly heterogenous disease, combining tumors with different locations and various genetic alterations, which can cause high variability in the performed experiments and complicates the detection of minor differences induced by HPV(+) and HPV(-) sEVs.

Incubation of iDCs with sEVs from HNSCC patients’ plasma increased expression of CCR7, a receptor which is involved in migration of DCs to the lymph nodes, where they present antigens to T cells (49). Overexpression of CCR7 on DCs was shown to increase their anti-tumor properties and increase their migratory ability towards draining lymph nodes (50), suggesting an activation of the anti-tumor response also through circulating plasma-sEVs from HNSCC patients. In line with this hypothesis, DCs were reported to take up TAAs during initial cancer development and present these to T cells to orchestrate an anti-tumor immune response (51, 52). Hence, immature DCs seem to respond to TEX and HNSCC sEVs by promoting an anti-tumor response, underscoring their potential as a cancer vaccine strategy in personalized medicine.

However, these partially activated iDCs seem to be functionally impaired, as indicated by low or partial production of IL12. Furthermore, the expression levels of APM components in iDCs were lower compared to mature DCs (mDCs), which is to be expected and consistent with the literature (53, 54). This upregulation during maturation leads to a more effective interaction of DCs and T cells, resulting in the better recognition of tumor cells by T cells. Thus, lower expression of APM proteins indicates functional defects of DCs and potentially a lower ability to induce tumor-specific immune responses (23). In our study, incubation with TEX or HNSCC sEVs led to increased expression of activation markers in iDCs and partially increased production of IL12, but did not increase expression of APM components, maybe due to the lack of further co-stimulatory signals necessary for successful DC activation, suggesting functional defects in iDCs. In contrast, mature DCs were fully functional regardless of incubation with TEX.

TEX-mediated changes in DCs seem to be stronger in early stages of DC maturation and differentiation. In line with this, it has been reported that the APM components in monocyte-derived DCs from cancer patients are not up-regulated to the same extent as in monocytes from HDs during *ex vivo* maturation (23), suggesting a cancer-dependent inhibition of DC differentiation and maturation occurs already in monocytes. Furthermore, incubation of DCs with EVs from melanoma patients increased production of TGF-β, IL-6 and TNF-α, leading to immunosuppressive activity of DCs (39). A subset of these TGF-β producing, immunosuppressive cells was also found in increased numbers within the PBMCs of melanoma patients, further supporting the notion of impaired DC activation in cancer. Monocytes used in our experiments originated from healthy donors and were not exposed to other tumor-related factors besides the sEVs isolated from cancer patients or cell lines, indicating TEX as a major factor for DC activation.

Interestingly, incubation of mDCs with HNSCC sEVs decreased levels of APM components, which is associated with loss of DC function. It has been shown that the ability to process and cross-present exogenous antigens is impaired in DCs with a higher lipid load (55, 56). Lipids are also a part of sEVs and by internalizing sEVs, lipids are also internalized and might accumulate in DCs, causing impaired antigen processing. Another possible mechanism, by which EVs inhibit DC activation and functionality is through transfer of miRNAs, present in tumor-derived EVs. MiR-212-3p, for example, was shown to be transferred from pancreatic carcinoma-derived EVs to DCs, where it decreased MHC-II expression levels, leading to immune tolerance (36). In an independent study performed by our group, miRNA present in samples of plasma-derived sEVs from healthy donors and HNSCC patients were measured and revealed upregulation of miR-212-3p in HNSCC patients compared to HD sEVs. Consistent with the findings of Ding et al., miR-212-3p may also contribute to the functional impairment of DCs we observed in our experiments.

In conclusion, unlike cell line-derived TEX, which influence iDC activation within the TME, sEVs from HNSCC patients’ plasma suppress the APM and hinder the induction of an anti-tumor response in the periphery, with miR-212-3p being the potential driver of this observation.

HNSCC sEVs, especially HPV(+) TEX, seem to activate immature DCs to induce an anti-tumor immune response, however, sEV incubation failed to fully activate iDCs and might cause functional defects instead. In contrast, mature DCs are less responsive and are inhibited by HNSCC sEVs through downregulation of the APM components. This observation is important also regarding *ex vivo* DC activation, since during immature stages, sEVs seem to increase anti-tumor response, while incubation of mature DCs leads to inhibition of DCs.

The incomplete activation of sEV-treated iDCs might be overcome by addition of maturation cytokines after sEV incubation. However, in the context of DC-based vaccines, this implies that *in vitro*-activated DCs can lose functionality upon exposure to circulating sEVs in the plasma of HNSCC patients, potentially compromising the efficacy of this therapeutic approach. *In vivo*, sEVs not only inhibit the DC-mediated anti-tumor response, but were also shown to interfere with immune therapies, including therapeutic antibodies, and mediate resistance to chemotherapies (37). DCs are important targets for immune therapies and combining several therapeutic approaches, including depletion of immunosuppressive sEVs, re-activation of DCs and inhibition of immune checkpoints, can be beneficial. For example, immune checkpoint blockers are only effective in patients with pre-existing antitumor immunity and functional DCs (4, 7, 51, 57). Additional administration of oncolytic viruses or TLR receptor agonists were able to reactivate plasmacytoid DCs to exert cytotoxic functions and turn immunologically cold tumors into hotspots of inflammation (58, 59). Considering the importance of functional DCs for a specific anti-tumor response, as well as new immunotherapeutic approaches, the effects of TEX on DC functions should be further investigated. Moreover, approaches to bypass the sEV-transmitted inhibitory effects on mature DCs should be addressed in the future, as well as possible implication to use HNSCC plasma sEVs or sEVs from tumor explants as source for TAAs to activate tumor-specific DCs *ex vivo* and develop new personalized therapy approaches.

## Data Availability

The original contributions presented in the study are included in the article/**Supplementary Material**. Further inquiries can be directed to the corresponding author/s.

## References

[1] Johnson DE Burtness B Leemans CR Lui VWY Bauman JE Grandis JR . Head and neck squamous cell carcinoma. Nat Rev Dis Primers. (2020) 6:92. doi: 10.1038/s41572-020-00224-3, PMID: 33243986 PMC7944998

[2] Pulte D Brenner H . Changes in survival in head and neck cancers in the late 20th and early 21st century: A period analysis. Oncologist. (2010) 15:994–1001. doi: 10.1634/theoncologist.2009-0289, PMID: 20798198 PMC3228039

[3] Benites BD Alvarez MC Saad STO . Small particles, big effects: the interplay between exosomes and dendritic cells in antitumor immunity and immunotherapy. Cells. (2019) 8:1648. doi: 10.3390/cells8121648, PMID: 31888159 PMC6952774

[4] Broz ML Binnewies M Boldajipour B Nelson AE Pollack JL Erle DJ . Dissecting the tumor myeloid compartment reveals rare activating antigen-presenting cells critical for T cell immunity. Cancer Cell. (2014) 26:638–52. doi: 10.1016/j.ccell.2014.09.007, PMID: 25446897 PMC4254577

[5] Spranger S Dai D Horton B Gajewski TF . Tumor-residing batf3 dendritic cells are required for effector T cell trafficking and adoptive T cell therapy. Cancer Cell. (2017) 31:711–723.e4. doi: 10.1016/j.ccell.2017.04.003, PMID: 28486109 PMC5650691

[6] Saddawi-Konefka R O'Farrell A Faraji F Clubb L Allevato MM Jensen SM . Lymphatic-preserving treatment sequencing with immune checkpoint inhibition unleashes cDC1-dependent antitumor immunity in HNSCC. Nat Commun. (2022) 13:4298. doi: 10.1038/s41467-022-31941-w, PMID: 35879302 PMC9314425

[7] Kono M Saito S Rokugo M Egloff AM Uppaluri R . Enhanced oral versus flank lymph node T cell response parallels anti-PD1 efficacy in head and neck cancer. Oral Oncol. (2024) 152:106795. doi: 10.1016/j.oraloncology.2024.106795, PMID: 38599127 PMC11065458

[8] Wculek SK Cueto FJ Mujal AM Melero I Krummel MF Sancho D . Dendritic cells in cancer immunology and immunotherapy. Nat Rev Immunol. (2020) 20:7–24. doi: 10.1038/s41577-019-0210-z, PMID: 31467405

[9] Steinman RM . Decisions about dendritic cells: past, present, and future. Annu Rev Immunol. (2012) 30:1–22. doi: 10.1146/annurev-immunol-100311-102839, PMID: 22136168

[10] Ebstein F Kloetzel P-M Krü E Seifert U . Emerging roles of immunoproteasomes beyond MHC class I antigen processing. Cell Mol Life Sci. (2012) 69:2543–58. doi: 10.1007/s00018-012-0938-0, PMID: 22382925 PMC11114860

[11] López-Albaitero A Mailliard R Hackman T Andrade Filho PA Wang X Gooding W . Maturation pathways of dendritic cells determine TAP1 and TAP2 levels and cross-presenting function. J Immunotherapy. (2009) 32:465–73. doi: 10.1097/CJI.0b013e3181a1c24e, PMID: 19609238 PMC2913548

[12] Schuler PJ Börger V Bölke E Habermehl D Matuschek C Wild CA . Dendritic cell generation and CD4+CD25highFOXP3 + regulatory T cells in human head and neck carcinoma during radio-chemotherapy. Eur J Med Res. (2011) 16:57–62. doi: 10.1186/2047-783X-16-2-57, PMID: 21463982 PMC3353422

[13] Kacani L Wurm M Schwentner I Andrle J Schennach H Sprinzl GM . Maturation of dendritic cells in the presence of living, apoptotic and necrotic tumour cells derived from squamous cell carcinoma of head and neck. Oral Oncol. (2005) 41:17–24. doi: 10.1016/j.oraloncology.2004.05.006, PMID: 15598581

[14] Lee KW Yam JWP Mao X . Dendritic cell vaccines: A shift from conventional approach to new generations. Cells. (2023) 12:2147. doi: 10.3390/cells12172147, PMID: 37681880 PMC10486560

[15] Zhang HG Grizzle WE . Exosomes and cancer: A newly described pathway of immune suppression. Clin Cancer Res. (2011) 17:959. doi: 10.1158/1078-0432.CCR-10-1489, PMID: 21224375 PMC3155407

[16] Bobrie A Colombo M Raposo G Théry C . Exosome secretion: molecular mechanisms and roles in immune responses. Traffic. (2011) 12:1659–68. doi: 10.1111/j.1600-0854.2011.01225.x, PMID: 21645191

[17] da Costa VR Araldi RP Vigerelli H D'Ámelio F Mendes TB Gonzaga V . Exosomes in the tumor microenvironment: from biology to clinical applications. Cells. (2021) 10:2617. doi: 10.3390/cells10102617, PMID: 34685596 PMC8533895

[18] Ludwig S Floros T Theodoraki MN Hong CS Jackson EK Lang S . Suppression of lymphocyte functions by plasma exosomes correlates with disease activity in patients with head and neck cancer. Clin Cancer Res. (2017) 23:4843. doi: 10.1158/1078-0432.CCR-16-2819, PMID: 28400428 PMC5559308

[19] Whiteside TL . The effect of tumor-derived exosomes on immune regulation and cancer immunotherapy. Future Oncol. (2017) 13:2583. doi: 10.2217/fon-2017-0343, PMID: 29198150 PMC5827821

[20] Beccard IJ Hofmann L Schroeder JC Ludwig S Laban S Brunner C . Immune suppressive effects of plasma-derived exosome populations in head and neck cancer. Cancers (Basel). (2020) 12:1997. doi: 10.3390/cancers12071997, PMID: 32708274 PMC7409343

[21] Theodoraki M Hoffmann TK Whiteside TL . Separation of plasma-derived exosomes into CD3 (1) and CD3 (-) fractions allows for association of immune cell and tumour cell markers with disease activity in HNSCC patients. Clin Exp Immunol. (2018) 192:271–283. doi: 10.1111/cei.13113, PMID: 29431869 PMC5980445

[22] Schroeder JC Puntigam L Hofmann L Jeske SS Beccard IJ Doescher J . Circulating exosomes inhibit B cell proliferation and activity. Cancers (Basel). (2020) 12:2110. doi: 10.3390/cancers12082110, PMID: 32751214 PMC7464446

[23] Whiteside TL Stanson J Shurin MR Ferrone S . Antigen-processing machinery in human dendritic cells: up-regulation by maturation and down-regulation by tumor cells 1. J Immunol. (2004) 173:1526–34. doi: 10.4049/jimmunol.173.3.1526, PMID: 15265880

[24] Ludwig S Sharma P Theodoraki MN Pietrowska M Yerneni SS Lang S . Molecular and functional profiles of exosomes from HPV(+) and HPV(–) head and neck cancer cell lines. Front Oncol. (2018) 8:445. doi: 10.3389/fonc.2018.00445, PMID: 30370252 PMC6194188

[25] Muller L Hong CS Stolz DB Watkins SC Whiteside TL . Isolation of biologically-active exosomes from human plasma. J Immunol Methods. (2014) 411:55. doi: 10.1016/j.jim.2014.06.007, PMID: 24952243 PMC4260336

[26] Théry C Witwer KW Aikawa E Alcaraz MJ Anderson JD Andriantsitohaina R . Minimal information for studies of extracellular vesicles 2018 (MISEV2018): a position statement of the International Society for Extracellular Vesicles and update of the MISEV2014 guidelines. J Extracell Vesicles. (2018) 7:1535750. doi: 10.1080/20013078.2018.1535750, PMID: 30637094 PMC6322352

[27] Hofmann L Ludwig S Schuler PJ Hoffmann TK Brunner C Theodoraki MN . The potential of CD16 on plasma-derived exosomes as a liquid biomarker in head and neck cancer. Int J Mol Sci. (2020) 21:3739. doi: 10.3390/ijms21113739, PMID: 32466374 PMC7312379

[28] Hofmann L Abou Kors T Ezić J Niesler B Röth R Ludwig S . Comparison of plasma- and saliva-derived exosomal miRNA profiles reveals diagnostic potential in head and neck cancer. Front Cell Dev Biol. (2022) 10. doi: 10.3389/fcell.2022.971596, PMID: 36072342 PMC9441766

[29] Wang X Campoli M Cho HS Ogino T Bandoh N Shen J . A method to generate antigen-specific mAb capable of staining formalin-fixed, paraffin-embedded tissue sections. J Immunol Methods. (2005) 299:139–51. doi: 10.1016/j.jim.2005.02.006, PMID: 15896802

[30] Ogino T Wang X Ferrone S . Modified flow cytometry and cell-ELISA methodology to detect HLA class I antigen processing machinery components in cytoplasm and endoplasmic reticulum. J Immunol Methods. (2003) 278:33–44. doi: 10.1016/S0022-1759(03)00224-2, PMID: 12957394

[31] Ogino T Wang X Kato S Miyokawa N Harabuchi Y Ferrone S . Endoplasmic reticulum chaperone-specific monoclonal antibodies for flow cytometry and immunohistochemical staining. Tissue Antigens. (2003) 62:385–93. doi: 10.1034/j.1399-0039.2003.00114.x, PMID: 14617045

[32] Kaliński P Smits HH Schuitemaker JH Vieira PL van Eijk M de Jong EC . IL-4 is a mediator of IL-12p70 induction by human th2 cells: reversal of polarized th2 phenotype by dendritic cells. J Immunol. (2000) 165:1877–81. doi: 10.4049/jimmunol.165.4.1877, PMID: 10925267

[33] Mailliard RB Wankowicz-Kalinska A Cai Q Wesa A Hilkens CM Kapsenberg ML . alpha-type-1 polarized dendritic cells: A novel immunization tool with optimized CTL-inducing activity. Cancer Res. (2004) 64:5934–7. doi: 10.1158/0008-5472.CAN-04-1261, PMID: 15342370

[34] Lane P Burdet C McConnell F Lanzavecchia A Padovan E . CD40 ligand-independent B cell activation revealed by CD40 ligand-deficient T cell clones: evidence for distinct activation requirements for antibody formation and B cell proliferation. Eur J Immunol. (1995) 25:1788–93. doi: 10.1002/eji.1830250646, PMID: 7615009

[35] Vieira PL de Jong EC Wierenga EA Kapsenberg ML Kaliński P . Development of th1-inducing capacity in myeloid dendritic cells requires environmental instruction. J Immunol. (2000) 164:4507–12. doi: 10.4049/jimmunol.164.9.4507, PMID: 10779751

[36] Ding G Zhou L Qian Y Fu M Chen J Chen J . Pancreatic cancer-derived exosomes transfer miRNAs to dendritic cells and inhibit RFXAP expression via miR-212-3p. Oncotarget. (2015) 6:29877–88. doi: 10.18632/oncotarget.4924, PMID: 26337469 PMC4745769

[37] Hosseini R Asef-Kabiri L Yousefi H Sarvnaz H Salehi M Akbari ME . The roles of tumor-derived exosomes in altered differentiation, maturation and function of dendritic cells. Mol Cancer. (2021) 20:83. doi: 10.1186/s12943-021-01376-w, PMID: 34078376 PMC8170799

[38] Xiang X Poliakov A Liu C Liu Y Deng ZB Wang J . Induction of myeloid-derived suppressor cells by tumor exosomes. Int J cancer J Int du Cancer. (2009) 124:2621. doi: 10.1002/ijc.24249, PMID: 19235923 PMC2757307

[39] Valenti R Huber V Filipazzi P Pilla L Sovena G Villa A . Human tumor-released microvesicles promote the differentiation of myeloid cells with transforming growth factor-β–mediated suppressive activity on T lymphocytes. Cancer Res. (2006) 66:9290–8. doi: 10.1158/0008-5472.CAN-06-1819, PMID: 16982774

[40] Ning Y Shen K Wu Q Sun X Bai Y Xie Y . Tumor exosomes block dendritic cells maturation to decrease the T cell immune response. Immunol Lett. (2018) 199:36–43. doi: 10.1016/j.imlet.2018.05.002, PMID: 29800589

[41] Ludwig S Marczak L Sharma P Abramowicz A Gawin M Widlak P . Proteomes of exosomes from HPV(+) or HPV(-) head and neck cancer cells: differential enrichment in immunoregulatory proteins. Oncoimmunology. (2019) 8:1593808. doi: 10.1080/2162402X.2019.1593808, PMID: 31143515 PMC6527282

[42] Ludwig S Sharma P Wise P Sposto R Hollingshead D Lamb J . mRNA and miRNA profiles of exosomes from cultured tumor cells reveal biomarkers specific for HPV16-positive and HPV16-negative head and neck cancer. Int J Mol Sci. (2020) 21:1–15. doi: 10.3390/ijms21228570, PMID: 33202950 PMC7698015

[43] Schrank TP Prince AC Sathe T Wang X Liu X Alzhanov DT . NF-κB over-activation portends improved outcomes in HPV-associated head and neck cancer. Oncotarget. (2022) 13:707. doi: 10.18632/oncotarget.28232, PMID: 35634245 PMC9131933

[44] Shi S Wang L Wang C Xu J Niu Z . Serum-derived exosomes function as tumor antigens in patients with advanced hepatocellular carcinoma. Mol Immunol. (2021) 134:210–7. doi: 10.1016/j.molimm.2021.03.017, PMID: 33819783

[45] Xu Z Zeng S Gong Z Yan Y . Exosome-based immunotherapy: a promising approach for cancer treatment. Mol Cancer. (2020) 19:160. doi: 10.1186/s12943-020-01278-3, PMID: 33183286 PMC7661275

[46] Liu H Chen L Peng Y Yu S Liu J Wu L . Dendritic cells loaded with tumor derived exosomes for cancer immunotherapy. Oncotarget. (2017) 9:2887–94. doi: 10.18632/oncotarget.20812, PMID: 29416821 PMC5788689

[47] Theodoraki MN Hoffmann TK Whiteside TL . Separation of plasma-derived exosomes into CD3(+) and CD3(–) fractions allows for association of immune cell and tumour cell markers with disease activity in HNSCC patients. Clin Exp Immunol. (2018) 192:271–83. doi: 10.1111/cei.13113, PMID: 29431869 PMC5980445

[48] Yan W Jiang S . Immune cell-derived exosomes in the cancer-immunity cycle. Trends Cancer. (2020) 6:506–17. doi: 10.1016/j.trecan.2020.02.013, PMID: 32460004

[49] Ohl L Mohaupt M Czeloth N Hintzen G Kiafard Z Zwirner J . CCR7 governs skin dendritic cell migration under inflammatory and steady-state conditions. Immunity. (2004) 21:279–88. doi: 10.1016/j.immuni.2004.06.014, PMID: 15308107

[50] Okada N Mori N Koretomo R Okada Y Nakayama T Yoshie O . Augmentation of the migratory ability of DC-based vaccine into regional lymph nodes by efficient CCR7 gene transduction. Gene Ther. (2005) 12:129–39. doi: 10.1038/sj.gt.3302358, PMID: 15483669

[51] Brandum EP Jørgensen AS Rosenkilde MM Hjortø GM . Dendritic cells and CCR7 expression: an important factor for autoimmune diseases, chronic inflammation, and cancer. Int J Mol Sci. (2021) 22(15):8340. doi: 10.3390/ijms22158340, PMID: 34361107 PMC8348795

[52] Jhunjhunwala S Hammer C Delamarre L . Antigen presentation in cancer: insights into tumour immunogenicity and immune evasion. Nat Rev Cancer. (2021) 21:298–312. doi: 10.1038/s41568-021-00339-z, PMID: 33750922

[53] Macagno A Kuehn L de Giuli R Groettrup M . Pronounced up-regulation of the PA28alpha/beta proteasome regulator but little increase in the steady-state content of immunoproteasome during dendritic cell maturation - PubMed. Eur J Immunol. (2001) 31:3271–80. doi: 10.1002/1521-4141(200111)31:11<3271::AID-IMMU3271>3.0.CO;2-2, PMID: 11745344

[54] Li J Schuler-Thurner B Schuler G Huber C Seliger B . Bipartite regulation of different components of the MHC class I antigen-processing machinery during dendritic cell maturation. Int Immunol. (2001) 13:1515–23. doi: 10.1093/intimm/13.12.1515, PMID: 11717192

[55] Ramakrishnan R Tyurin VA Veglia F Condamine T Amoscato A Mohammadyani D . Oxidized lipids block antigen cross-presentation by dendritic cells in cancer. J Immunol. (2014) 192:2920–31. doi: 10.4049/jimmunol.1302801, PMID: 24554775 PMC3998104

[56] Herber DL Cao W Nefedova Y Novitskiy SV Nagaraj S Tyurin VA . Lipid accumulation and dendritic cell dysfunction in cancer. Nat Med. (2010) 16:880–6. doi: 10.1038/nm.2172, PMID: 20622859 PMC2917488

[57] Appleton E Hassan J Chan Wah Hak C Sivamanoharan N Wilkins A Samson A . Kickstarting immunity in cold tumours: localised tumour therapy combinations with immune checkpoint blockade. Front Immunol. (2021) 12:754436. doi: 10.3389/fimmu.2021.754436, PMID: 34733287 PMC8558396

[58] Schuster P Lindner G Thomann S Haferkamp S Schmidt B . Prospect of plasmacytoid dendritic cells in enhancing anti-tumor immunity of oncolytic herpes viruses. Cancers (Basel). (2019) 11:651. doi: 10.3390/cancers11050651, PMID: 31083559 PMC6562787

[59] Kim Y Clements DR Sterea AM Jang HW Gujar SA Lee PW . Dendritic cells in oncolytic virus-based anti-cancer therapy. Viruses. (2015) 7:6506–25. doi: 10.3390/v7122953, PMID: 26690204 PMC4690876

