# Supplementary material for: Differential effects of small extracellular vesicles from head and neck cancer patients on dendritic cell functions
Source: Front Oncol. 2026 Jan 14;15:1680167. doi: 10.3389/fonc.2025.1680167 (PMC12846954; doi:10.3389/fonc.2025.1680167)
Supplement: Supplementary file 1 [file DataSheet1.docx]

Supplementary Material


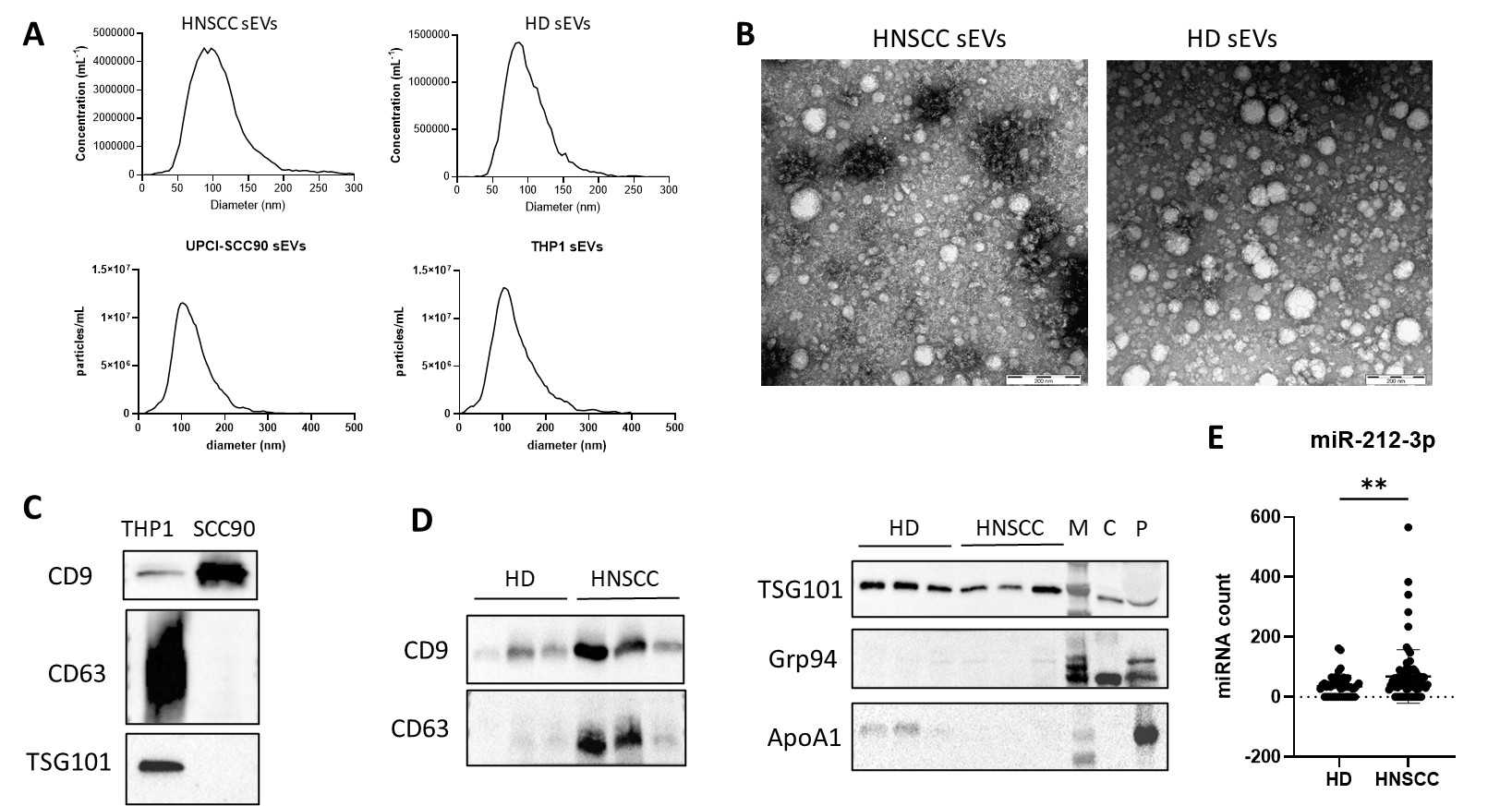


**Supplementary Figure 1.** Characterization of plasma-derived sEVs according to MISEV Guidelines. **A**: Size distribution of representative sEV samples derived from plasma of HNSCC patients or healthy donors (upper row) or from cell culture supernatants of THP-1 cells or UPCI-SCC90 (lower row) determined by nanoparticle tracking analysis with Zeta View. **B:** Representative transmission electron microscopy (TEM) images of sEV from HNSCC patients (left**)** or healthy donors (right). **C+D**: Western blots of sEV markers present in sEVs samples (CD9, CD63, TSG101) and absence of plasma (ApoA1) and cell (Grp94) markers of cell line-derived sEVs (**C**) and plasma-derived sEVs (**D**). **E**: Counts of miR-212-3p in a cohort of n = 49 HD and n = 80 HNSCC patients. Individual values with mean and SD. P-value determined by Mann-Whitney test. HNSCC: Head and neck squamous cell carcinoma patient-derived sEVs; HD: healthy donor derived sEVs; M: protein ladder; C: cell lysate as control for negative marker Grp94; P: plasma sample as control for negative marker ApoA1.

**Supplementary Figure 2**: Comparison of effect of HPV-positive and HPV-negative patient-derived sEVs in immature DCs. Expression activation and maturation markers (**A**) or APM components (**B**) after incubation with sEVs derived from plasma of HPV-positive (HPV+ sEVs) and HPV-negative (HPV- sEVs) HNSCC patients or healthy donors (HD sEVs) on day 0 and day 3, measured on day 6. The data are from 3 experiments performed with DCs generated from monocytes of 3 healthy donors, incubated with sEVs from 12 HNSCC patients, 6 for each group of HPV-positive or HPV negative and 3 healthy donors. Differences with statistical significance were indicated with stars above the respective comparison with * representing p < 0.05; **: p < 0.01 and ***: p < 0.005. All other comparisons were not significant.

**Supplementary Figure 3**: Comparison of effect of HPV-positive and HPV-negative patient-derived sEV in mature DCs. Expression activation and maturation markers (**A**) or APM components (**B**) after incubation with sEVs derived from plasma of HPV-positive (HPV+ sEVs) and HPV-negative (HPV- sEVs) HNSCC patients or healthy donors (HD sEVs) on day 7, measured on day 8. The data are from 3 experiments performed with DCs generated from monocytes of 3 healthy donors, incubated with sEVs from 12 HNSCC patients, 6 for each group of HPV-positive or HPV negative and 3 healthy donors. Differences with statistical significance were indicated with stars above the respective comparison with * representing p < 0.05; **: p < 0.01 and ***: p < 0.005. All other comparisons were not significant.
